# Toxic Bradycardias in the Critically Ill Poisoned Patient

**DOI:** 10.1155/2012/852051

**Published:** 2012-04-01

**Authors:** Melissa L. Givens

**Affiliations:** Department of Emergency Medicine, Carl R. Darnall Army Medical Center, Fort Hood, TX 76544-4752, USA

## Abstract

Cardiovascular drugs are a common cause of poisoning, and toxic bradycardias can be refractory to standard ACLS protocols. It is important to consider appropriate antidotes and adjunctive therapies in the care of the poisoned patient in order to maximize outcomes. While rigorous studies are lacking in regards to treatment of toxic bradycardias, there are small studies and case reports to help guide clinicians' choices in caring for the poisoned patient. Antidotes, pressor support, and extracorporeal therapy are some of the treatment options for the care of these patients. It is important to make informed therapeutic decisions with an understanding of the available evidence, and consultation with a toxicologist and/or regional Poison Control Center should be considered early in the course of treatment.

## 1. Background

Nearly 2000 poisoned patients are seen per day in Emergency Departments across the United States, and unintentional poisoning is a significant cause of mortality even surpassing motor vehicle accidents as a cause of death in people aged 35–54 [1]. Cardiovascular drugs rank second only behind analgesics as the leading cause of fatality in poisoned patients. Polypharmacy, intentional or unintentional ingestions, and toxic exposures should be entertained in the differential diagnosis of the bradycardic critically ill patient. Consideration and recognition of poisoning may shed light onto altered physiologic responses that may be refractory to traditional therapies. Standard resuscitation algorithms are often insufficient, and it is important to consider appropriate antidotes and adjunctive therapies when caring for the poisoned patient. Additionally, consultation with a toxicologist or poison control center is recommended to assist in caring for the poisoned patient. 

Toxic bradycardias are often refractory to standard ACLS protocols due to toxin effects on cardiac and vascular receptors and cellular physiology. Recognition of a toxic etiology for compromised circulation in the setting of bradycardia is crucial in tailoring appropriate therapy. Beta blockers, calcium channel blockers, and cardiac glycosides (digoxin) represent the classes of medication most described in association with fatality due to drug exposure according to the American Association of Poison Control Centers. This discussion will also briefly cover clonidine and acetylcholinesterase inhibitors, such as organophosphates and carbamates, because both have therapeutic consideration outside of standard supportive care.

This paper discusses common treatment considerations that apply to the critically ill poisoned patient with a toxic bradycardia. The goal is to focus on the evidence or lack of evidence for specific therapies but not to provide an exhaustive review of each toxin and/or medication. A MEDLINE search was conducted using the following search terms: Beta blocker OR beta antagonist, calcium channel blocker OR calcium antagonist, clonidine, digoxin, acetylcholinesterase inhibitor OR organophosphate OR carbamate; overdose OR toxicity; insulin, glucagon, calcium chloride OR calcium gluconate, lipid emulsion OR intralipid, vasopressors, epinephrine, norepinephrine, dopamine, vasopressin, atropine, Pralidoxime OR 2-PAM, naloxone, Digibind OR DigiFab, balloon pump, CVVHD, ECMO, and cardiopulmonary bypass. Case reports, case series, and human and animal studies pertinent to the etiologies of bradycardia discussed hereinafter were included. Laboratory studies, studies targeting specific organs or tissue, and cases with multiple substances ingested were excluded. Major toxicology textbooks were also reviewed for expert consensus.

While this paper highlights current literature, it is important to remember that toxicology research is often limited to case series, case reports, and animal studies with few controlled trials. Many treatment guidelines are based on expert consensus, and further research is encouraged to strengthen an evidence-based approach to the care of the poisoned patient.

## 2. Decontamination

Decontamination is a complex and controversial issue that is an important consideration in every poisoned patient. A complete discussion of decontamination is beyond the scope of this review but key points related to decontamination of the patient with toxic bradycardia will be highlighted. Standard gastrointestinal decontamination techniques include orogastric lavage (OGL), activated charcoal (AC), and whole bowel irrigation (WBI). Despite popular application of these techniques for poisoned patients in the past, none of these therapies has proven to have a significant impact on clinical outcomes and, thus, their use has been largely limited to specific situations. Because ingestion of cardioactive drugs often is associated with significant morbidity and mortality, early aggressive decontamination may be of relative benefit.

For patients who present within 1 hour of ingestion of a potentially serious toxin, AC or OGL can be considered if airway protection can be ensured [2, 3]. However, many cardiovascular drugs come in formulations that may be too large to pass through the holes of a lavage tube and this should be considered when deciding to perform OGL. WBI may be considered for patients who present with ingestion of medications that are sustained release or extended release preparations. Often calcium channel blockers and beta blockers come in SR or XL forms that may be amenable to WBI [4]. 

## 3. Therapy

### 3.1. Beta Blockers and Calcium Channel Blockers

Calcium channel blockers and beta blockers are separate categories of medication each with their own distinct mechanism of action, but it is very difficult to distinguish the two classes of medication in a patient who has overdosed. In the absence of a good history or pill bottle evidence, a clinician may have to treat a patient based on the assumption that the offending agent is either a calcium channel blocker or beta blocker. Common findings include myocardial depression and peripheral vasodilation. AV conduction abnormalities, idioventricular rhythms, and heart block may be seen on EKG. Most calcium channel blockers and the lipophilic beta blockers undergo hepatic metabolism and liver failure can result in accumulation, while the water soluble forms can easily accumulate in patients with renal failure. Toxicity can be profound when the two classes of agents are coingested. Fortunately the treatment of both classes of medication overlaps and that is why they are discussed together. Special note should be given to the beta blockers with membrane stabilizing effects and potassium channel blockade, as therapy needs to be tailored in consideration of these additional mechanisms of toxicity.

 While there is a paucity of literature that directly addresses the efficacy of specific antidotes in the setting of beta blocker and calcium blocker toxicity, there are some small studies and anecdotal evidence to guide therapy in patients who do not respond to standard ACLS protocols. The International Liaison Committee on Resuscitation in conjunction with the American Heart Association recently published novel guidelines on the resuscitation of poisoned patients [5].

 There are numerous case studies showing improvement of hemodynamic parameters without significant adverse effects for beta-blocker-poisoned patients treated with glucagon [6–15]. Glucagon bypasses the beta receptor to initiate the same intracellular cascade as a beta agonist. A bolus dose of 5–10 mg followed by an infusion of 1–5 mg/hr (0.15 mg/kg) is recommended. It is important to treat the patient with an antiemetic prior to glucagon infusion, as nausea and vomiting are a common side effect.

 High-dose insulin-euglycemic therapy (HIE) also shows promise in both beta-blocker- and calcium-channel-blocker-induced hemodynamic compromise based on case reports and animal data [16–33]. The exact mechanism has yet to be elucidated but is believed to be related to improved metabolic function of the cell. While hypoglycemia is a concern, proper monitoring and glucose supplementation will prevent iatrogenic injury. Regular insulin can be given as an initial 0.5–2.0 unit bolus followed by 0.5 units per hour with glucose supplementation and electrolyte monitoring with special attention given to potassium. 

 Calcium infusion, while long considered the mainstay of therapy in calcium channel blocker overdose, only has anecdotal evidence to support its use and there is no clear consensus on appropriate dosing [34, 35]. An appropriate starting dose is 1–3 gm, and higher doses have been used with success in refractory cases. Calcium gluconate (10%) is preferred when given through a peripheral line and proper dosing is 3 times the equivalent volume of calcium chloride (10%) [36].

 Lipid emulsion therapy is a novel therapy originally directed at anesthetic toxicity but there are animal studies and case reports in beta blocker and calcium channel blocker overdose that indicate a potential benefit [37–40].

 Bradycardia with decreased perfusion caused by digoxin or plants and herbal medications with cardiogenic glycosides should be treated with antidigoxin immune fragments. Dosing can be delivered empirically, based on measured digoxin levels, or based on known ingestion amounts. See Table 1 for dosing guidelines. Hyperkalemia is an ominous finding in digoxin toxicity and serves as an indicator of toxicity. Avoidance of calcium salts in the setting of digoxin poisoning has been a long-standing axiom. This mantra is based on the infamous “stone heart” phenomena described in dogs in 1939 [41]. However, there is literature to refute this consequence when calcium was given to digoxin-poisoned swine [42]. Despite this evidence, there is no role for calcium in the setting of hyperkalemia due to digoxin poisoning, but this study can provide reassurance for the clinician who wants to give calcium in cases of undifferentiated bradycardia that is unlikely but not certain to be digoxin toxicity. Evidence shows that treatment of the hyperkalemia does not improve outcomes, and it is more important to pursue antibody treatment of the underlying digoxin toxicity [43]. Although part of standard ACLS protocols, cardiac pacing can be detrimental in digoxin-poisoned patients. Higher morbidity and mortality has been described in digoxin-poisoned patients who underwent pacing compared with those who received immunotherapy alone [44]. Class Ib antidysrhythmics such as phenytoin or lidocaine can be used as temporizing therapy until immune fragments are available. Phenytoin has been shown to improve digitalis-induced AV nodal conduction blockade [45, 46].

Clonidine may also cause hemodynamically significant bradycardia. Most cases of clonidine overdose respond well to supportive care; however refractory cases may occur. There is mixed success reported in the literature regarding the use of naloxone for clonidine-induced bradycardia and hypotension, even when used at higher doses [47–50]. Aggressive supportive care and standard ACLS therapy with fluid resuscitation, atropine, and pressor support is usually adequate for clonidine poisoned patients. Yohimbe can be considered as an alpha-2 antagonist but the benefit is theoretical and delivery is limited to nonpharmaceutical grade oral preparations [51].

 For bradycardia caused by acetylcholinesterase inhibitors, atropine is the mainstay of therapy, not only for the bradycardia but more importantly to treat the copious bronchial secretions due to cholinergic excess. A useful starting dose is 2 mg but gram-quantity doses may be required [52, 53]. Early mobilization of pharmacy resources is necessary to ensure that adequate stocks of atropine are available. Concurrent airway management is paramount, and endotracheal intubation with positive pressure ventilation should be considered early. Use of neuromuscular blocking agents that are metabolized by cholinesterase such as succinylcholine may result in prolonged paralysis and alternate agents should be utilized [54]. Pralidoxime should be given as an adjunct to atropine in patients exposed to organophosphates to prevent aging and to provide relief from both muscarinic and nicotinic symptoms [55, 56]. The initial dose is 1-2 grams IV over 10–15 minutes followed by an infusion of 250–500 mg/hour. Pralidoxime use in the setting of carbamates is controversial, and consultation with a toxicologist is recommended [57].

### 3.2. Pressor Support

Despite ACLS recommendations to consider pressor therapy in poisoned patients with hemodynamic compromise, there is no clear consensus regarding the best choice of medication for hemodynamic support. There are numerous studies exploring various pressors and combinations in the setting of many different toxins. There are serious limitations in all the available literature and no agent can be considered superior.  Table 2 highlights the available studies related to vasopressor therapy in the toxic agents discussed previously. Attention to cardiac output and peripheral vascular resistance can help guide the choice of pharmacologic agents for hemodynamic support.

### 3.3. Extracorporeal Therapy

While a mainstay of ACLS protocols, cardiac pacing may have limited utility in the patient with toxic bradycardia due to beta blockers or calcium channel blockers and may even be detrimental in patient with digoxin toxicity as noted previously. Pacing may improve heart rate, but even if there is electrical capture, improvement in hemodynamic parameters may not be seen [58–62]. There are a variety of other therapeutic options that are mentioned in the literature without rigorous study to support or refute their efficacy [62–67]. Consideration of nonpharmaceutical therapies such as CVVHD, intra-aortic balloon pumps, and ECMO should be considered on a case by case basis utilizing the expertise of the appropriate consultant.

## 4. Conclusion

ACLS protocols may be of limited utility when treating poisoned patients with toxic bradycardia. Use of specific antidotes/adjunctive therapies may prove helpful and should be considered early in the course of treatment in consultation with a medical toxicologist or regional poison control center. 

## Figures and Tables

**Table 1:** Digoxin antibody dosing recommendations.

Known amount ingested	Amount of digoxin ingested (mg)/0.5 = number of vials
Known serum level	((Serum digoxin (ng/mL) × weight(kg)))/100 = number of vials

Empiric dosing	Acute overdose = 10 vials
	Chronic overdose = 5 vials

**Table 2:** Literature on vasopressor use in toxic bradycardia.

Study	Summary
Kerns, W., 2nd, et al., Insulin improves survival in a canine model of acute beta-blocker toxicity. Annals of Emergency Medicine, 1997. 29(6): p. 748–57.	Survival better for animals treated with insulin compared to those treated with glucagon or epinephrine.

Toet, A.E., et al., Reduced survival after isoprenaline/dopamine in d,l-propranolol intoxicated rats. Human and Experimental Toxicology, 1996. 15(2):120–8.	No improvement in hemodynamic variables with isoproterenol. Addition of dopamine resulted in decreased MAP and survival time.

Toet, A.E., et al., Experimental study of the detrimental effect of dopamine/glucagon combination in d,l-propranolol intoxication. Human and Experimental Toxicology, 1996. 15(5): 411–21.	No improvement of survival time with dopamine/glucagon but some improvement in hemodynamic variables.

Holger, J.S., et al., A comparison of vasopressin and glucagon in beta-blocker induced toxicity. Clinical Toxicology: The Official Journal of the American Academy of Clinical Toxicology and European Association of Poisons Centres and Clinical Toxicologists, 2006. 44(1): 45–51.	Vasopressin resulted in higher MAP/SBP but no difference in survival compared to glucagon in porcine model.

Holger, J.S., et al., Insulin versus vasopressin and epinephrine to treat beta-blocker toxicity. Clinical Toxicology: The Official Journal of the American Academy of Clinical Toxicology and European Association of Poisons Centres and Clinical Toxicologists, 2007. 45(4): 396–401.	Increased SVR with vasopressin/epinephrine but decreased survival when compared to insulin in porcine model.

Kanagarajan, K., et al., The use of vasopressin in the setting of recalcitrant hypotension due to calcium channel blocker overdose. Clinical Toxicology: The Official Journal of the American Academy of Clinical Toxicology and European Association of Poisons Centres and Clinical Toxicologists, 2007. 45(1): p. 56–9.	Successful use of vasopressin in patient refractory to other therapies.

Kline, J.A., E. Leonova, and R.M. Raymond, Beneficial myocardial metabolic effects of insulin during verapamil toxicity in the anesthetized canine. Critical Care Medicine, 1995. 23(7): p. 1251–63.	Insulin resulted in improved hemodynamic variables compared to epinephrine, calcium chloride, or glucagon.

Stone, C.K., et al., Glucagon and phenylephrine combination versus glucagon alone in experimental verapamil overdose. [see comment]. Academic Emergency Medicine, 1996. 3(2): p. 120–5.	Decreased survival when phenylephrine combined with glucagon.

Barry, J.D., et al., Vasopressin treatment of verapamil toxicity in the porcine model. J Med Toxicol, 2005. 1(1): p. 3–10.	Decreased survival with vasopressin.

Sztajnkrycer, M.D., et al., Use of vasopressin in a canine model of severe verapamil poisoning: a preliminary descriptive study. Academic Emergency Medicine, 2004. 11(12): p. 1253–61.	Decreased cardiac index and no improvement in MAP.

Anderson FJ, Hart GR, Crumpler CP, Lerman MJ: Clonidine overdose: Report of six cases and review of the literature. Ann Emerg Med 1981; 10 : 107–112.	Successful use of dopamine for improved blood pressure.
